# Construct Validity and Reliability of a New Spanish Empathy Questionnaire for Children and Early Adolescents

**DOI:** 10.3389/fpsyg.2017.00979

**Published:** 2017-06-13

**Authors:** Maria C. Richaud, Viviana N. Lemos, Belen Mesurado, Laura Oros

**Affiliations:** ^1^Consejo Nacional de Investigaciones Científicas y TécnicasBuenos Aires, Argentina; ^2^Centro de Investigaciones en Psicología y Ciencias Afines, Universidad Adventista del PlataLibertador San Martin, Argentina

**Keywords:** multidimensional empathy, new questionnaire, evaluation, psychometrics, validity, childhood

## Abstract

Empathy is a basic socio-emotional process of human development that involves the ability to perceive, share, and understand the emotional states of others. This process is essential to successful social functioning. However, despite its significance, empathy has been difficult to define and measure, particularly when incorporating both its emotional and cognitive aspects. The purpose of this study was to develop an Empathy Questionnaire for children aged 9–12 years based on a model of social cognitive neuroscience and to analyze its construct validity and reliability. This questionnaire aimed to integrate the following aspects: *emotional contagion, self-other awareness, perspective-taking, emotional regulation*, and *empathic action. Three* studies were conducted. Study 1 evaluated the discriminative power of the items and studied the underlying structure of the instrument using exploratory factor analysis. In Study 2, confirmatory factor analysis was performed to test the model obtained. Finally, the goal of Study 3 was to analyze the convergent and discriminant validity of the questionnaire and the internal consistency of its dimensions. The final version of the instrument contained 15 items that operationalized the previously listed dimensions. The results of the 3 studies indicated that the questionnaire had good validity and reliability. This study has important implications for research and clinical practice. Given its simplicity and brevity, this new self-report scale may work well as a screening method to evaluate the key psychological issues underlying numerous child behaviors that predict the success or failure of social relationships, individual quality of life, and mental well-being.

## Introduction

Empathy is an indispensable skill for human beings, whose lives are based on complex social contexts. The social nature of human beings is such that the recognition and understanding of the mental states of others and the ability to share these mental states and adequately respond to them are equally or more important than the understanding of and response to non-social natural contexts (López et al., 2014). This social and emotional competence underlies some of the most important human interactions, from the early bonding between mother and child to other complex prosocial behaviors (Batson, 2009).

Considering the significant role that empathy plays in human development, it is essential to assess and measure it to identify when its development has been altered and when to intervene. Recent advances in social cognitive neuroscience have indicated that empathy, as an inductive process, can be described and thus be measured. This realization has led to significant implications for its conceptualization (Decety and Moriguchi, 2007; Decety and Meyer, 2008). Thus, the objective of this article is to construct a five-dimensional questionnaire of empathy for children 9–12 years based in cognitive neuroscience developments.

According to Bensalah et al. (2016) to date, four questionnaires have been used to assess empathy in middle childhood (Bryant, 1982; Litvack-Miller et al., 1997; Garton and Gringart, 2005; Reid et al., 2013). Garton and Gringart's (2005) questionnaire studies the role of empathy in collaborative problem solving in school education. Garton and Gringart's (2005) and Litvack-Miller et al.'s (1997) questionnaires, used with children aged 7–9 or 7–11 years, respectively, are independent adaptations of the Interpersonal Reactivity Index (IRI; Davis, 1980). The Index of Empathy (Bryant, 1982) for children aged 6–12 years only assess emotional empathy as a vicarious emotional response to the emotion of another person. Reid and colleagues developed the Kids' Empathic Development Scale (Reid et al., 2013). This scale assesses empathy in 7–11 year-olds that have to answer to emotional stories. It considers three factors: an affective component, a cognitive component referring to the justifications that children produce to explain their emotional inference, and a behavioral component that concerns adaptive behaviors in relation to the emotional scenario (Bensalah et al., 2016). Additionally, the Empathy Questionnaire for Children and Adolescents (Pouw et al., 2013) operationalizes the affective aspects of empathy.

Finally, Bensalah et al. (2016) have adapted for French children aged 6–11 years, the adult French version of the Basic Empathy Scale (Carré et al., 2013) of a 20-item scale developed by Jolliffe and Farrington (2006) for adolescents. Bensalah et al. postulate a three-factor model of empathy: emotional contagion, cognitive empathy, and emotional disconnection. Except in the latter case, which is based on three primary aspects of empathy suggested by Decety and Moriguchi (2007), the scales mentioned before do not offer a measure of empathy that reflect the understanding of empathy based in neuroscience.

Some current models have integrated the *automatic* or *unconscious emotional experience* of the observed or inferred emotional state with the recognition and understanding of the emotional states of others through controlled *cognitive processes*. Decety and Jackson (2004), for example, present a model that accounts for the affective and cognitive components of empathy. They argue that reflecting the emotions of others is a basic process that can occur without the intervention of consciousness, whereas processes that characterize human empathy, such as perspective taking and self-regulation, require controlled processing.

Empathy not only involves the emotional experience of another person's emotional state or emotional contagion, whether real or inferred, but also the recognition and a minimal understanding of the emotional state of the other person. This definition captures the multidimensional nature of empathy and explicitly references a minimum capacity to mentalize (Decety and Moriguchi, 2007). Some authors suggest that there is neural basis that supports a model of two separate systems for empathy: an emotional system and a cognitive system (Shamay-Tsoory, 2011; Cox et al., 2012).

Hoffman (2000) suggested empathy might represent a developmental process, progressing from automatic mimicry (Iacoboni, 2009) to cognitive processing that involves the ability to imagine the experiences of another. In the same line, Decety and Jackson (2004) demonstrated that observable brain activity was associated with four subjectively experienced components of empathy: affective sharing, self-awareness, perspective taking, and emotion regulation. Decety and Moriguchi's (2007) descriptions of these four components increased the clarity of the overall conceptualization of empathy (Lietz et al., 2011).

Recent research has suggested a fifth potentially important component of empathy (Lietz et al., 2011). Gerdes and Segal (2009) have presented a 3-dimensional diagram based on the developments of Decety et al. (Decety and Jackson, 2004; Decety and Lamm, 2006). This model includes three components: (1) the affective response to the emotions and actions of others, (2) the cognitive processing of the other's perspective and one's own affective response, and (3) the conscious decision making to undertake an empathic or prosocial action that Eisenberg (2006, p. 71) called *empathy related responding*, which would explain the relationship between empathy and prosocial behavior. According to this model, the empathic awareness of another's discomfort (e.g., pain, distress) should lead to solidarity or altruism (Lietz et al., 2011).

In view of the theoretical considerations above, this study aimed to design a self-report instrument that would obtain a multidimensional measure of empathy for 9–12 year-old children based on the model of Decety and Jackson (2004). The model would include the empathic action dimension suggested by Gerdes and Segal (2009). Accordingly, the objective was to operationalize the following dimensions of empathy in children: *emotional contagion, self-other awareness, perspective-taking, emotional regulation*, and *empathic action*, questionnaire integrates affective and cognitive components of empathy. According to our best knowledge, there is no other questionnaire to measure empathy in children based on Decety and Moriguchi's (2007) four components model and including empathic action.

The measurement of the empathy construct is challenging, particularly for children. In addition to children's known difficulties in responding to questionnaires (i.e., less attention, language development) is the complexity of the dimensions to be implemented and the development of items for children that have content validity (Sartori and Pasini, 2007; Lietz et al., 2011). The items referred to self-awareness, for example, are very complex since the child must simultaneously look for what he felt at the moment of affective excitement (Lamm et al., 2007) and then suspend his own experience to evoke the thoughts and feelings of others. In turn, this skill is a prerequisite for perspective taking, which is a similar but distinct process because it involves that the other is different and yet to be put in their place. Emotional regulation implies changing the way people think which in turn changes the way they feel—a complex cognitive process (Ochsner et al., 2002; Lietz et al., 2011). Finally, when dealing with children, these complex processes must be expressed in short sentences with the least possible logical complexity.

We have chosen this stage of development in children because all the empathic dimensions proposed have already developed, although some of them continue to mature through late childhood and adolescence. Between 18 and 72 h after birth, newborns who hear other children cry often show distress reactions, suggesting an automatic or reactive contagion-type mechanism (Simner, 1971; Sagi and Hoffman, 1976; Martin and Clark, 1982). During the second year of life, throughout the development of the self-other differentiation, perspective-taking, and emotional regulation, a transformation from self-concern to concern for others occurs (Knafo et al., 2008). By 2 years of age, children begin to exhibit the basic behaviors of empathy by having emotional responses that correspond to other people's emotional states and by manifesting more organized prosocial behaviors (e.g., helping, sharing, or comforting) indicative of concern for others (Hoffman, 2000; Decety and Jackson, 2004). In the preschool years (4–5 years old), children are capable of taking the perspective of the other in pretend tasks that are frequently used as indicators of the development of a theory of mind (Wimmer and Perner, 1983; Wellman et al., 2001). Growth in the ability to identify oneself with the experience of others permits children between 7 and 12 years of age to show a natural inclination to feel empathy for others who suffer pain (Decety et al., 2008) and to develop more effective helping strategies (McDonald and Messinger, 2011). Surtees and Apperly (2012) found that perspective taking increases during the middle childhood period. Children's progress in emotional understanding is due to improvements both in making inferences about others' mental states and in attentional, memory, and inhibitory control abilities. Emotion regulation skills develop throughout childhood and adolescence, gradually allowing children to control emotion, affect, motivation, and urges (Bensalah et al., 2016). The mastery of emotional regulation occurs gradually across an extended period of childhood (e.g., Zimmer-Gembeck and Skinner, 2011). Cognitive control becomes more efficient between 8 and 12 years of age (Choudhury et al., 2006) and continues to improve between the ages of 12–13 and 19 years (Keulers et al., 2010; Vetter et al., 2013; Van der Graaff et al., 2014). Children aged 6–10 begin to understand that there are social group's informal norms about when, where, and how one should express emotions (Siegler, 2006). They come to appreciate the contexts in which certain emotional expressions are socially most appropriate and therefore ought to be regulated (Harris, 1983). This capacity of emotional self-regulation is related to sympathy or the affective aspect of empathy. Adequately self-regulated children would be expected to experience others' emotions vicariously yet not become overly aroused and overwhelmed by the emotion (Eisenberg, 2000).

## Procedures for the development of the scale items

The items were developed based on the theoretical models proposed by Decety and Jackson (2004) and by Gerdes and Segal (2009). The expressions used in each item were clear and simple and employed typical expressions for Argentine children aged 9–12 years.

Alternative expressions were suggested for some items and experts on the subject selected the best option. Thus, a preliminary scale of 50 items was formed, 10 for each dimension, which was presented to a sample of 17 children (9 girls and 8 boys) evenly representing different ages (4 children aged 9, 4 children aged 10, 5 children aged 11, and 4 children aged 12). The children were asked to assess the clarity of the instructions and the response options as well as the level of difficulty of the terms or expressions used in each sentence. Based on the children's recommendations, the instructions and the response options remained intact, but the items received revisions. Twelve statements were changed to get clearer expressions (e.g., “I find it easy to see the point of view of others” was replaced by “I find it easy to understand other people's different ways of thinking”); others items were excluded because they involved a level of abstraction too complex for this age group (e.g., “I can distinguish what I feel from what another person is feeling”), and a new one was proposed for the emotional regulation scale (e.g., “When I get angry, I find it difficult to calm down”).

The questionnaire resulting from the previous revision comprised 47 items: 7 on *emotional contagion* (e.g., “When I'm with someone who is sad, I also feel sad”); 10 on *self-awareness*, as different from the other (e.g., “Even though I am happy, I notice when a friend is angry”); 10 on *perspective-taking* (e.g., “Although others think differently, I can understand them”); 11 on *emotional regulation* (e.g., “When I get angry I find it extremely difficult to calm down”); and 9 on *empathic action* (e.g., “We must share with those who have less than us”).

## Instrument study

### Study 1

The purpose of the first study was to evaluate the items' psychometric performance and to study the underlying structure of the instrument to assess the multidimensional construct of empathy in children.

#### Methods

##### Participants

Parental informed consent and voluntary consent of the children was received prior to participation. An intentional, non-probabilistic sample of 205 school children (112 girls, 54.6%) from the Entre Rios, Misiones, and Buenos Aires provinces was used. Children were in the fifth (39%), sixth (36.6%), and seventh (24.4%) grades of five elementary schools participating in the first study. Mean age was 11.14 years (SD = 1.19; rank 9–12 years old). The children belonged to middle socioeconomic status, being the distribution of the mother's educational level was: 0% primary education, 40% secondary education, and 60% with tertiary or higher educational studies and distribution of the father's educational level was: 0% primary education, 35% secondary education, and 75% with tertiary or higher educational studies. The distribution of the occupation of the head of the family was: 15% qualified manual labor, 20% administrative and sales, and 65% university-level professionals, financiers, business people, all high productivity.

##### Instruments

The instrument administered was the preliminary version of the multidimensional Empathy Questionnaire for children. It comprised 47 items with four response options: always, often, sometimes, and never.

##### Procedures

The discriminative power of the items was examined using a contrasting groups method; the children's responses were analyzed using a *t*-test to identify the difference in means for independent samples. This analysis determined whether there were statistically significant differences between children with higher empathy scores and those with lower scores (upper quartile or 75th percentile and lower quartile or 25th percentile). A Principal Component Analysis (PCA) was run to investigate the dimensionality of the instrument.

#### Results

All of the items were discriminative (*p* < 0.001; See Table 1).

**Table 1:** Discriminative power of the items in the final version of the scale.

**Items de la escala**	**Low group**		**High group**		***t***
	***M***	***SD***	***M***	***SD***	
1. When I see someone crying who I do not know, I feel like crying.	1.55	0.77	2.58	0.93	5.94^***^
5. When I see someone dancing, I feel like moving my feet.	2.30	1.15	3.37	0.87	5.02^***^
8. When I am with someone who is sad, it makes me feel sad too.	2.00	0.94	3.28	0.88	6.81^***^
2. I immediately notice when someone feels bad.	2.87	0.96	3.86	0.41	6.78^***^
13. I notice when a friend is bored.	3.08	0.91	3.77	0.48	4.75^***^
9. Even though I am happy, I notice when a friend is angry.	2.87	1.05	3.93	0.25	7.06^***^
14. When I argue with someone, I try to understand what he or she is thinking.	2.15	1.01	3.58	0.58	8.68^***^
6. Even though another person may think differently, I can understand him/her.	2.23	0.89	3.79	0.46	11.05^***^
3. I find it easy to understand other people's different ways of thinking.	2.25	0.80	3.14	0.88	5.16^***^
4. I have fits of anger.	2.06	1.16	3.14	1.01	4.79^***^
7. When I get angry, I find it difficult to calm down.	1.74	0.96	3.16	0.94	7.18^***^
11. I change all of the time; at times I feel good, and suddenly, I get angry.	2.36	1.16	3.07	1.10	3.05^**^
10. If a child forgets his/her pencil case, I should lend him/her my school things.	2.92	1.07	3.67	0.68	4.16^***^
12. We must share with those who have less than us.	3.21	0.84	3.98	0.15	6.53^***^
15. I think we all must help children in need.	3.08	0.87	4.00	0.00	7.70^***^

** *p ≤ 0.01*,

*** *p ≤ 0.001*.

The value of the *Kaiser-Meyer-Olkin* measure of Sampling Adequacy was 0.759, and the Bartlett's test of sphericity was χ^2^ = 452.12; *p* < 0.001.

When defining the factors, only items weighing 0.40 or more were considered. After discarding factorially complex items, that is those with a similar weighing on more than one factor and those weighing less than the predetermined value in the corresponding factor, a simple structure consisting of 15 items was obtained. This structure comprised three items per factor that were answered on a 4-point scale (from always to never) and accounted for 55.46% of the variance (see Table 2). The McDonald's omega coefficients for each factor are shown at Table 2.

**Table 2:** Results of the Principal ComponentAnalysis, Factor Matrix, and Oblimin Rotation.

**Items**	**ER**	**EC**	**PT**	**SA**	**EA**
4. I have fits of anger^*^.	**0.706**				
7. When I get angry, I find it difficult to calm down^*^.	**0.682**				
11. I change all of the time; at times I feel good, and suddenly, I get angry^*^.	**0.726**				
1. When I see someone crying who I do not know, I feel like crying.		**0.803**			
5. When I see someone dancing, I feel like moving my feet.		**0.405**			
8. When I am with someone who is sad, it makes me feel sad too.		**0.822**			
14. When I argue with someone, I try to understand what he or she is thinking.			**0.562**		
3. Even though another person may think differently, I can understand him/her.			**0.736**		
6. I find it easy to understand other people's different ways of thinking.			**0.669**		
2. I immediately notice when someone feels bad.				**0.637**	
9. I notice when a friend is bored.				**0.726**	
13. Even though I am happy, I notice when a friend is angry.				**0.534**	
10. If a child forgets his/her pencil case, I should lend him/her my school things.					**−0.699**
12. We must share with those who have less than us.					**−0.705**
15. I think we all must help children in need.					**−0.649**
Mean	2.60	2.44	2.77	3.37	3.58
SD	0.59	0.74	0.70	0.59	0.52
Skewness	−0.17	0.14	−0.21	−0.92	−1.24
Kurtosis	−0.84	−0.60	−0.66	0.55	0.62
Rank (minimum and maximum)	1–4	1–4	1–4	1–4	1.67–4
McDonald's coefficient omega	0.75	0.73	0.70	0.67	0.73

*EC, Emotional Contagion; SA, Self-others Awareness; PT, Perspective-Taking; ER, Emotional Regulation; EA, Empathic Action*.

* *Reverse Items*.

*Bold values represent component weightings*.

### Study 2

This study aimed to test the model obtained in the first study through a confirmatory factor analysis (CFA).

#### Methods

##### Participants

Prior parental informed consent and voluntary participation of the children was obtained. The second study was performed with an intentional sample of 212 school children (119 girls, 55.9%) from the Entre Rios, Misiones, and Buenos Aires provinces who were in the fifth (32%), sixth (38%), and seventh (30%) grade of four elementary schools. Their mean age was 11.08 years (*SD* = 0.94; rank 9–12 years). The children belonged to the middle socioeconomic class, being the distribution of the mother's educational level was: 0% primary education, 37% secondary education, and 63% with tertiary or higher educational studies, and the father's educational level was: 0% primary education, 38% secondary education, and 62% with tertiary or higher educational studies. The distribution of the occupation of the head of the family was: 10% qualified manual labor, 25% administrative and sales, and 65% university-level professionals, financiers, business people, all high productivity.

##### Instruments

The instrument administered was the 15-item version of the multidimensional Empathy Questionnaire for children that resulted from study 1. In the present study, McDonald's omega was as follows: Emotional Contagion = 0.78, Self-others Awareness = 0.75, Perspective-Taking = 0.72, Emotional Regulation = 0.72, and Empathic Action = 0.70.

##### Procedures

To verify whether the data from a new study sample fit the structure found by the PCA performed in study 1, using Maximum Likelihood Estimator a CFA was conducted to assess the fit indices: GFI (Goodness of Fit Index), CFI (Comparative Fit Index), and NFI (Bentler-Bonett Normed Fit Index), and RMSEA (Root Mean Square Error of Approximation) to assess the degree of error of the model. The CFA was performed through AMOS 16.0 (Arbuckle, 2007).

#### Results

##### Construct validity

**Figure 1:**
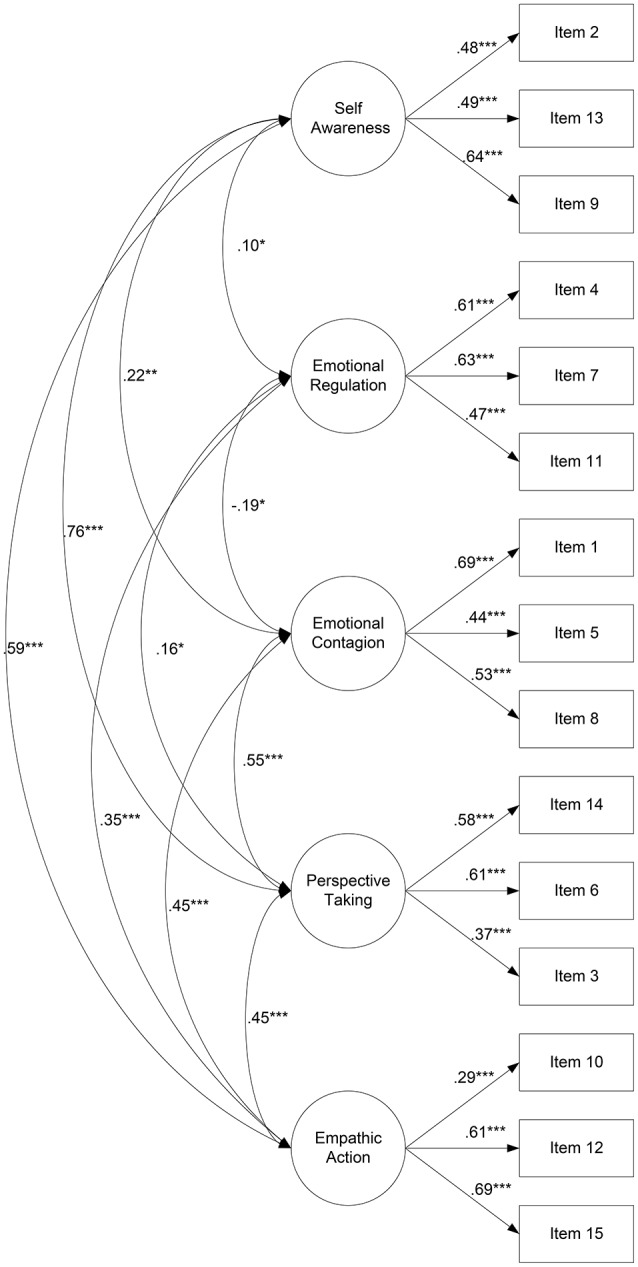
Five factor model of empathy. ^*^p ≤ 0.05, ^**^p ≤ 0.01, ^***^p ≤ 0.001.

The five-factor model fit the data very well, as we can see through the values of fit indexes: χ^2^(80) = 88.041, *p* = 0.252, χ^2^/*df* = 1.10; *GFI* = 0.95; *NFI* = 0.91, *CFI* = 0.98, and *RMSEA* = 0.02, and it is depicted in Figure 1.

### Study 3

The purpose of the third study was to verify the construct validity of the instrument by testing its convergent and discriminant validity and to analyze the internal consistency of the different dimensions of empathy.

Several studies found that individuals who frequently participated in prosocial behaviors were more empathic and showed less aggressive behaviors (Carlo et al., 1992, 2011; Eisenberg et al., 2006; Garaigordobil and De Galdeano, 2006). The dimensions of empathy that were evaluated by the new instrument were therefore hypothesized to have a positive relationship with prosociality and a negative relationship with physical and verbal aggression. Moreover, evidence exists about a negative relationship between *emotional instability* and the dimension of *emotional regulation* (Singh and Mishra, 2011; Khosla, 2012; Bujor and Turliuc, 2014). Thus, a negative relationship between these constructs was hypothesized. A positive correlation between *empathic action* and *prosocial behavior* was also expected to be observed, as well as a positive relationship between *perspective-taking* as assessed by the IRI (Davis, 1983) and the *perspective-taking* dimension of the new instrument.

#### Methods

##### Participants

A total of 479 schoolchildren (257 girls, 53.7%), in the fourth (19.4%), fifth (25.7%), sixth (29%), and seventh (25.9%) grades of four primary schools participated in this study. The participants were selected using a purposive sampling framework. Their mean age was 10.77 years (SD = 1.16; rank 9–12 years). The children were from the middle socioeconomic class, being the distribution of the mother's educational level: 0% primary education, 38% secondary education, and 62% with tertiary or higher educational studies, and of the father's educational level: 0% primary education, 35% secondary education, and 65% with tertiary or higher educational studies. The distribution of the occupation of the head of the family was: 12% qualified manual labor, 28% administrative and sales, and 60% university-level professionals, financiers, business people, all high productivity.

##### Instruments

In addition to the multidimensional instrument of empathy for children presented in studies 1 and 2, the following questionnaires were administered.

– The *Prosocial Behavior Scale* (PB, Caprara and Pastorelli, 1993, Spanish version by Del Barrio et al., 2001). Prosocial behavior encompasses all those voluntary acts that contribute to the benefit of other individuals (Eisenberg and Mussen, 1989; Roche Olivar, 2011). The PB is a scale composed of 15 items that evaluate the behavior of altruism, trust, and pleasantness through three response alternatives as a function of frequency with each of the behaviors described (often, sometimes, and never). This self-report provides an overall measure of child prosociality. The internal consistency obtained in this study was α = 0.79.
– The *Physical and Verbal Aggression Scale* (FVA, Caprara and Pastorelli, 1993; Spanish version by Del Barrio et al., 2001). This scale is a 20-item self-report questionnaire that evaluates physically and verbally behavior that is harmful to others. The response format consists of 3 options: often, sometimes, and never. In this study, the internal consistency of the scale was α = 0.73.
– The *Emotional Instability Scale* (EI, Caprara and Pastorelli, 1993; Spanish version by Del Barrio et al., 2001). This scale measures the lack of self-control in social situations as the result of a limited capacity to curb impulsiveness and emotionality. This instrument is self-reported and consists of 20 items. The questionnaire uses 3 response options: often, sometimes, and never. The internal consistency of the scale in this study was α = 0.83.
– The *Interpersonal Reactivity Index* (IRI, Davis, 1980). The IRI operationalize a cognitive and affective multidimensional empathy construct. IRI measures four dimensions: (a) *fantasy*, the tendency to identify with characters in films and in literature; in other words, it assesses the subject's imaginative capacity to place him or herself in fictitious situations, (b) *perspective-taking*, or the ability to understand another person's point of view, (c) *emotional distress*, feelings of anxiety and restlessness shown by the person when observing situations in which another person experiences negative experiences, and (d) *empathic concern*, feelings of compassion, concern, and care toward others. In the present study, we administered an adapted version for children of IRI carried out in Argentina (Richaud de Minzi, 2007). In this study, we only considered the dimension Perspective Taking, since the objective of this study was to evaluate the convergent validity of the new scale of empathy proposed in this work, and this dimension is the only one that would have a greater theoretical correspondence with it. In this study sample, the Cronbach's alpha was 0.64. The response format consists of 5 options: the statement describes me very well, well, somewhat, a little, and not at all.
– The *Multidimensional Scaling of Children Prosociality* (Lemos, 2015). This scale evaluates the following prosocial behaviors through 23 items: assistance, giving and sharing, comforting, positive confirmation of the other, and cooperation. The possible responses to the items are always, sometimes, rarely, and never. The internal consistency of the overall scale that was obtained for this study sample was α = 0.90.

##### Procedures

The instruments were administered collectively in two different situations to avoid fatigue of the children. Three scales were completed in the first session (Prosocial Behavior Scale, Physical and Verbal Aggression Scale, and Emotional Instability Scale) and two in the second (Interpersonal Reactivity Index and Multidimensional Scaling of Children Prosociality). The order of administration was maintained constant for all school groups.

To provide further evidence of the instrument's validity, Pearson's *r* correlations were performed to assess the strength and the direction of the relationship between the dimensions of empathy represented in the new Questionnaire and the following variables: IRI *perspective-taking, prosocial behavior, emotional instability*, and *physical and verbal aggression*.

#### Results

##### Convergent validity

Significant and positive correlations were observed between the results of the two prosocial behavior scales (Caprara and Pastorelli, 1993; Lemos, 2015) and the dimensions of the new Empathy Questionnaire: *emotional contagion, self-other awareness, perspective-taking, emotional regulation*, and *empathic action*. A significant positive correlation between *perspective-taking* assessed by the IRI and *perspective-taking* assessed by the new Empathy Questionnaire was also obtained (see Table 3).

**Table 3:** Correlations between emotional contagion, self-other awareness, perspective-taking, emotional regulation, and empathic action with prosocial behavior, emotional instability, aggression, and IRI perspective-taking.

	**Prosocial behavior Caprara and Pastorelli (1993)**	**Prosocial behavior Lemos (2015)**	**Emotional instability**	**Verbal and physical aggression**	**Perspective taking IRI**
EC	0.316^**^	326^**^	−0.024	−0.187^*^	0.321^**^
SA	0.382^**^	0.373^**^	−0.123	−0.190^*^	0.190^*^
PT	0.489^**^	0.452^**^	−0.121	−0.256^**^	0.373^**^
ER	0.228^**^	0.212^**^	−0.247^**^	−0.312^**^	0.026
EA	0.794^**^	0.497^**^	−0.146	−0.186^*^	0.359^*^

EC, Emotional Contagion; SA, Self-others Awareness; PT, Perspective-Taking; ER, Emotional Regulation; EA, Empathic Action.//

* *p ≤ 0.05*,

** *p ≤ 0.01*.

##### Discriminant validity

Significant negative correlations were found between the results obtained in the *physical and verbal aggression* scale and the *emotional contagion, self-other awareness, perspective-taking, emotional regulation*, and *empathic action* dimensions of the new Empathy Questionnaire. A significant negative correlation was also obtained between *emotional instability* and *emotional regulation* (see Table 3).

##### Reliability

The McDonald's omega of the dimensions were 0.75 for emotional contagion, 0.76 for self-other awareness, 0.72 for perspective-taking, 0.72 for emotional regulation, and 0.70 for empathic action. The individual item-total correlations for the five-factor structure are provided in Table 4.

**Table 4:** Item-total correlations.

**Item**	**EC**	**SA**	**PT**	**ER**	**EA**
1	0.58^***^				
5	0.64^***^				
8	0.57^***^				
2		0.52^***^			
9		0.23^**^			
13		0.52^***^			
14			0.60^***^		
6			0.55^***^		
3			0.44^***^		
4				0.44^***^	
7				0.63^***^	
11				0.52^***^	
10					0.39^***^
12					0.49^***^
15					0.41^***^

*EC, Emotional Contagion; SA, Self-others Awareness; PT, Perspective-Taking; ER, Emotional Regulation; EA, Empathic Action*.

*** *p = 0.001*.

## General discussion

The aim of the present study was to study the reliability and construct validity of a new empathy questionnaire for children of 9–12 years old.

As the concept of Emotional contagion appears very early in development (Decety and Jackson, 2004) the children were not expected to experience any difficulties responding to the corresponding items. A similar ease of responding was also expected for the items relating to *self-consciousness, self-other-awareness, perspective-taking, and emotional regulation*, processes that are expected to have developed at the age of study.

In the case of Empathic action, it is interesting to note this factor did not appear clearly in a previous study in which a preliminary version of the scale was developed (Richaud et al., 2015). Some of the empathic action items showed factorial complexity, while others had low factorial loads. Many of the items developed to operationalize empathic action involved a set of generalized beliefs and causal attributions about the misfortune of others as well as the determination of whether it was appropriate to assist them. For example, if it appears that the misfortunes of others are the result of their own voluntary decisions or their ineffectiveness (example: “It is wrong to give things to beggars; they ask for things because they do not want to work.”), the individual may be less likely to elicit empathic action. However, if the misfortune is attributed to causes beyond the control of the individual (example: “I think we all have to help kids in need”), the individual is more likely to have an empathic and caring disposition.

At first, this lack of clarity regarding the empathic action factor was attributed to a problem with its development, as the ability to enact an empathic action implied the internalization of certain values (e.g., solidarity, help, or altruism) that required the comprehension of abstract concepts not fully realized until adolescence. However, at 7 years old, children have already been shown to feel empathy for others suffering from pain (Decety et al., 2008) and accordingly to develop strategies of help and comfort.

Therefore, a better hypothesis may be that when items refer to a particular or a contextualized situation, individuals' responses may change and adapt based on their causal attribution to the situation. The contextual modulation of empathy makes an individual's behavior more sensitive to the conditions of different environments. To modify empathic actions according to the environment, our brains must access the available contextual information to predict the social meanings involved (e.g., others' intentions, feelings, and behavior) on the basis of previous experiences and the relevance of the particular situation (Hein and Singer, 2008; Singer and Lamm, 2009).

The relationship between empathy and prosociality has been well established (Bartal et al., 2011; Richaud, 2014; Williams et al., 2014). However, prosociality scales that contained general and not context-dependent statements (e.g., We must help those who suffer) were used in these studies. Because of these considerations, the authors of this study decided to retain items of empathic action on the scale that related to general situations to obtain the fifth dimension of the questionnaire.

In this study, an empathy scale for children was developed that was based on a multidimensional model that posits a series of stages in the development of empathy (Decety and Jackson, 2004; Decety and Moriguchi, 2007). In addition, the psychometric properties of the scale were analyzed. This study provides an original and significant contribution to the literature, as the operationalization of this model is unprecedented in Argentina.

### Strengths and limitations

The sound psychometric properties of the new scale confirm the notion that cognitive and affective aspects of empathy are closely intertwined. Accordingly, it would be inappropriate to exclude either of these aspects if the purpose is to achieve a comprehensive overview of the phenomenon of empathy. Based on the results obtained in this study, further analysis of the interactions between the five dimensions included in this model as well as their correlations with other processes not covered in this article may lead to a better understanding of the overall empathic experience.

This study has important implications for research and clinical practice. Given its simplicity and brevity, this new self-report scale may work well as a screening method to evaluate the key psychological issues underlying numerous child behaviors that predict the success or failure of social relationships, individual quality of life, and mental well-being.

The limitations of this study include the sampling criteria used for the selection of participants, as convenience sampling has several disadvantages compared to probability samples. Therefore, it may be appropriate to test the generalizability of these results in future studies by using samples that reflect a broader range of sociodemographic characteristics and a greater geographic diversity of the participants. The inclusion of a social desirability assessment is also recommended, as children have a tendency to provide socially acceptable answers to please others (Lemos, 2005, 2006); this is a general limitation of objective self-administered questionnaires (Lemos, 2013), so it would be advisable to complement the evaluation of the construct with other measures and informants (teachers, parents).

## Ethics statement

This study was carried out in accordance with the recommendations of APA Ethical Principles of Psychologists and Code of Conduct with written informed consent from all subjects. All subjects gave written informed consent in accordance with the Declaration of Helsinki. The protocol was approved by ethics committee of the Centro Interdisciplinario de Investigación Matemática y Experimental (CIIPME).

## Author contributions

MR designed the project, lead the data collection, wrote the Introduction and Discussion sections. VL and BM designed the project, lead the data collection, computed the analyses, wrote the Method, Results, and Discussion sections. LO lead the data collection and computed preliminary analyses.

### Conflict of interest statement

The authors declare that the research was conducted in the absence of any commercial or financial relationships that could be construed as a potential conflict of interest.
